# Supplementary material for: Hydroxyl-Group Identification Using O K-Edge XAFS in Porous Glass Fabricated by Hydrothermal Reaction and Low-Temperature Foaming
Source: Molecules. 2019 Sep 26;24(19):3488. doi: 10.3390/molecules24193488 (PMC6803955; doi:10.3390/molecules24193488)
Supplement: Supplementary file 1 [file molecules-24-03488-s001.pdf]

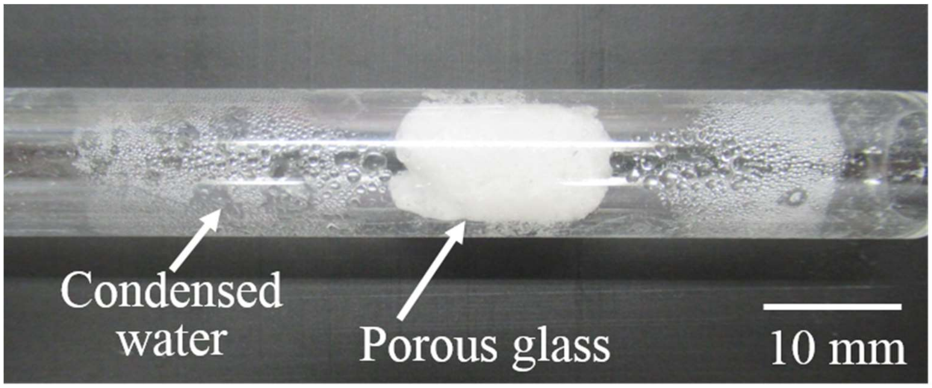

Figure 1. Porous glass expanded in a transparent glass tube, showing condensed water emitted from the hydrothermally treated glass when heated.

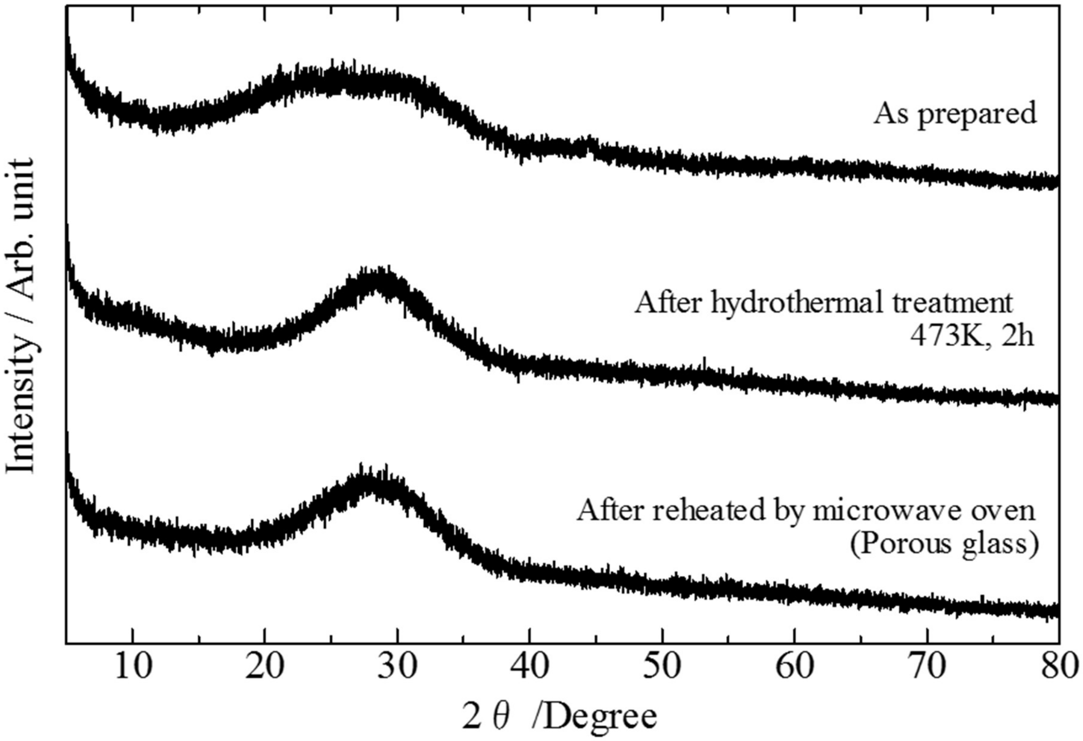

Figure 2. X-ray diffraction patterns of sodium borosilicate glass powders as prepared, after hydrothermal treatment and reheating by microwave oven (porous glass), where Cu Kα radiation was used.
